# Association of Mediterranean Diet With Cognitive Decline Among Diverse Hispanic or Latino Adults From the Hispanic Community Health Study/Study of Latinos

**DOI:** 10.1001/jamanetworkopen.2022.21982

**Published:** 2022-07-14

**Authors:** Bayan Moustafa, Gabriela Trifan, Carmen R. Isasi, Richard B. Lipton, Daniela Sotres-Alvarez, Jianwen Cai, Wassim Tarraf, Ariana Stickel, Josiemer Mattei, Gregory A. Talavera, Martha L. Daviglus, Hector M. González, Fernando D. Testai

**Affiliations:** 1 Department of Neurology and Rehabilitation, University of Illinois at Chicago College of Medicine, Chicago; 2 Department of Epidemiology and Population Health, Albert Einstein College of Medicine, Bronx, New York; 3 Department of Neurology, Albert Einstein College of Medicine, Bronx, New York; 4 Department of Biostatistics, Gillings School of Global Public Health, University of North Carolina, Chapel Hill; 5 Institute of Gerontology, Wayne State University, Detroit, Michigan; 6 Department of Neurosciences and Shiley-Marcos Alzheimer’s Disease Research Center, University of California San Diego, La Jolla; 7 Department of Nutrition, Harvard T.H. Chan School of Public Health, Boston, Massachusetts; 8 Department of Psychology, San Diego State University, San Diego, California; 9 Institute for Minority Health Research, University of Illinois at Chicago, Chicago

## Abstract

**Importance:**

The Mediterranean diet may reduce the burden of Alzheimer disease and other associated dementias in Hispanic or Latino people.

**Objective:**

To investigate the association of a Mediterranean diet with cognitive performance among community-dwelling Hispanic or Latino adults.

**Design, Setting, and Participants:**

This cohort study analyzed data from the Hispanic Community Health Study/Study of Latinos (HCHS/SOL) and the Study of Latinos–Investigation of Neurocognitive Aging (SOL–INCA), an HCHS/SOL ancillary study. Cognition tests were administered in the HCHS/SOL from March 2008 to June 2011 (visit 1) and in the SOL–INCA from October 2015 to March 2018 (visit 2). Participants included in the present study had completed a diet assessment at visit 1 and neurocognitive evaluations at visits 1 and 2. Data were analyzed from September 2021 to May 2022.

**Exposures:**

Mediterranean diet adherence was ascertained using the Mediterranean diet score (MDS) and was categorized as low (MDS: 0-4 points), moderate (MDS: 5-6 points), or high (MDS: 7-9 points). The mean of two 24-hour dietary recalls was used to calculate the MDS.

**Main Outcomes and Measures:**

Cognitive change between visits 1 and 2 was calculated by subtracting the cognitive score at visit 2 from the cognitive score at visit 1 and adjusting by the time elapsed between visits and cognitive score at visit 1. Neurocognitive tests administered were Brief Spanish-English Verbal Learning Test (B-SEVLT) Sum, B-SEVLT Recall, word fluency, and Digit Symbol Substitution Test (DSST). Results of each test were *z* score–transformed and the means were averaged to create a global cognition score. Complex sample linear regression analysis was used to ascertain the association between MDS and neurocognitive performance at each visit and neurocognitive change.

**Results:**

A total of 6321 participants (mean [SE] age, 56.1 [0.18] years at visit 1; n = 4077 women [57.8%]) were included. Mediterranean diet adherence weighted frequencies were 35.8% (n = 2112 of 6321) for the low adherence group, 45.4% (n = 2795) for the moderate adherence group, and 18.8% (n = 1414) for the high adherence group. In the fully adjusted model, *z* score–transformed cognitive scores at visit 1 in the high vs low adherence groups were higher for B-SEVLT Sum (β = 0.11; 95% CI, 0.02-0.20), B-SEVLT Recall (β = 0.16; 95% CI, 0.07-0.25), and global cognition (β = 0.10; 95% CI, 0.04-0.16) tests. In the mean follow-up time of 7 years, cognitive change in the high vs low adherence groups was less pronounced for B-SEVLT Sum (β = 0.12; 95% CI, 0.05-0.20) and B-SEVLT Recall (β = 0.14; 95% CI, 0.05-0.23), but not for word fluency, DSST score, or global cognition score.

**Conclusions and Relevance:**

Results of this cohort study suggested that high adherence to a Mediterranean diet was associated with better cognitive performance and decreased 7-year learning and memory decline among middle-aged and older Hispanic or Latino adults. Culturally tailored Mediterranean diet may reduce the risk of cognitive decline and Alzheimer disease in this population.

## Key Points

**Question:**

Is a Mediterranean diet associated with reduced cognitive decline among diverse Hispanic or Latino adults?

**Findings:**

In this cohort study of 6321 Hispanic or Latino adults, a high level of adherence to the Mediterranean diet was associated with better global cognition and decreased 7-year learning and memory decline compared with a low level of adherence to the diet.

**Meaning:**

Findings suggest that consumption of a culturally tailored Mediterranean diet may reduce the risk of cognitive decline and Alzheimer disease among middle-aged and older adults with Hispanic or Latino ethnicity.

## Teaser

This cohort study evaluates the association of reported consumption of Mediterranean-diet types of foods and beverages with cognitive performance changes among participants in the Hispanic Community Health Study/Study of Latinos and the Study of Latinos–Investigation of Neurocognitive Aging.

## Introduction

Cognitive impairment and dementia constitute a major public health challenge. Epidemiologic studies have demonstrated that cardiovascular risk factors are associated with cognitive decline and that their modification might prevent or delay the progression of at least 40% of dementias worldwide.^1^ The adoption of healthy lifestyle habits has been associated with lower risk of dementia, even among individuals with high genetic risk for dementia. Hispanic or Latino people, the fastest growing ethnic group in the US, have a substantial burden of Alzheimer disease and other associated dementias.^2,3^ In addition, it has been estimated that, by 2060, the number of Hispanic or Latino individuals with Alzheimer disease may increase by approximately 832% compared with 2012.^4^ Therefore, evaluating the potential risk factors and emerging preventive strategies in this group is crucial. Hispanic or Latino people, however, have been underrepresented in dementia research.^3,5^

The Mediterranean diet has been associated with decreased mortality and favorable cardiovascular and cognitive profiles.^6,7,8^ Previous studies found an association between adherence to a Mediterranean diet and lower cognitive impairment, even after adjusting for other risk factors.^9,10^ In addition, there is growing evidence of the benefits of the Mediterranean diet for cerebral structural integrity and neurodegeneration.^11,12^ In this longitudinal population-based cohort study, we aimed to investigate the association of a Mediterranean diet with cognitive performance among community-dwelling Hispanic or Latino adults. We hypothesized that higher adherence to a Mediterranean diet would be associated with decreased cognitive decline.

## Methods

This population-based cohort study was approved by the institutional review board at each of the participating sites. Written informed consent was obtained from participants before study initiation. We followed the Strengthening the Reporting of Observational Studies in Epidemiology (STROBE) reporting guideline.

### Study Sample

We analyzed data from the Hispanic Community Health Study/Study of Latinos (HCHS/SOL), a multisite, prospective, population-based cohort study of Hispanic or Latino adults aged 18 to 74 years living in the US. A stratified, 2-state area probability sample of household addresses was selected from 4 large US communities: San Diego, California; Chicago, Illinois; Bronx, New York; and Miami, Florida. Hispanic or Latino ethnicity was self-reported by participants and included Cuban, Central and South American, Mexican, Puerto Rican and Dominican. Participants aged 45 to 74 years were oversampled. Thus, sampling weights were used in the analysis to account for the unequal probability of selection. We also obtained data from the Study of Latinos–Investigation of Neurocognitive Aging (SOL–INCA), an ancillary study of the HCHS/SOL that investigated cognitive aging and impairment. Details of the HCH/SOL and SOL–INCA have been described previously.^13,14^

### Variables

Neurocognitive tests were administered in HCH/SOL and SOL–INCA during face-to-face encounters by trained bilingual psychometrists in the participants’ preferred language. Participants in HCH/SOL underwent neurocognitive assessments between March 2008 and June 2011 (visit 1). Participants who were recruited into SOL–INCA (n = 6377) underwent neurocognitive reassessment between October 2015 and March 2018 (visit 2). In the present study, we analyzed the results of 4 of these neurocognitive tests: (1) Brief Spanish-English Verbal Learning Test (B-SEVLT) Sum, which includes 3 learning trials, (2) B-SEVLT Recall, which is a delayed verbal episodic and memory trial; (3) word fluency (verbal) for speech production; and (4) Digit Symbol Substitution Test (DSST) for sustained attention, psychomotor speed, and executive function. These tests have been described previously.^15^

At visit 1, participants completed a 24-hour dietary recall of predefined food and nutritional categories. A second dietary recall was performed approximately 30 days later. For participants with 2 dietary recalls, dietary intake was ascertained by calculating the mean of both recalls. Adherence to a Mediterranean diet was examined using the Mediterranean diet score (MDS), which has been described previously.^8^ Briefly, the consumption of different MDS components (fruits, vegetables, legumes, cereals, fish, meat, and dairy products) was recorded and adjusted for total energy intake. Participants received 1 point for each beneficial component (fruits, vegetables, legumes, cereals, and fish) consumed at or above the sex-specific median, 1 point for each detrimental component (meat and dairy products) consumed below the sex-specific median, 1 point for monounsaturated to saturated fat ratio consumed above the sex-specific median, and 1 point for moderate alcohol consumption (≤2 drinks/d for men, and ≤1 drink/d for women). The mean servings for each MDS component are shown in Table 1.^8,16^ The MDS ranges from 0 to 9 points, with higher scores indicating greater adherence to a Mediterranean diet. In some Hispanic or Latino populations, the intake of refined grains and potatoes, which may not be fully beneficial, may be high.^17^ A modified MDS, which replaces the total-grain group with whole grains and excludes potato products from the vegetable group, has been proposed for this population.^16^

**Table 1.** Mediterranean Diet Score Components, Serving Size Definitions, and Energy-Adjusted Mean Servings at Visit 1 (N = 6321)^8,16^

Component	Mean (95% CI)	Definition
Beneficial components, servings/d		
Fruits	4.00 (3.89-4.11)	½-c (120 mL) or 1 medium piece of citrus and noncitrus fruit, or ½-c (120 mL) of juice
Legumes	0.87 (0.85-0.90)	42 g of nuts or 2 tbsp (10 mL) of nut butters or seeds, or ½-c (120 mL) of legumes
Cereals	6.17 (6.11-6.23)	Greater than sex-specific energy-adjusted median intake of whole grains (servings/d)
Fish	3.70 (3.67-3.72)	28 g of fish or shellfish
Vegetables	2.16 (2.13-2.20)	1 c (240 mL) of raw leafy vegetables or ½-c (120 mL) of other cooked or raw vegetables (dark-green, deep-yellow, other starchy vegetables), or ½-c (120 mL) of juice
Detrimental components, servings/d		
Meat	3.07 (2.99-3.15)	28 g of red or processed meat
Dairy products	1.62 (1.60-1.64)	1 c (240 mL) of milk or yogurt or 42 g of natural cheese or 52 g of processed cheese
Mild-to-moderate alcohol consumption, drinks/d	0.22 (0.19-0.25)	112 g of wine or 336 g of beer or 42 g of liquor
Monounsaturated-to-saturated fat (ratio)	1.15 (1.14-1.16)	

In this study, we used the original MDS and the modified MDS.^16,18^ Participants included in this analysis completed at least 1 dietary recall and neurocognitive assessments at visits 1 and 2.

### Covariates

Sociodemographic and clinical variables were ascertained at visit 1. Age, sex, household income, educational level, nativity (US-born vs non–US-born), preferred language, health insurance status, history of stroke or transient ischemic attack (TIA), and current or previous smoking status were self-reported by participants.

Blood pressure was measured with an automatic sphygmomanometer while the participant was seated. Three blood pressure measurements were obtained 1 minute apart, and the mean of these 3 values was used in this analysis. History of hypertension was defined as blood pressure higher than 140/90 mm Hg or receiving treatment for hypertension. Glycated hemoglobin A_1c_, total cholesterol, high-density lipoprotein cholesterol, low-density lipoprotein cholesterol, and creatinine and cystatin C levels were measured on a fasting blood sample. We used the Chronic Kidney Disease Epidemiology Collaboration creatinine–cystatin C equation to calculate the estimated glomerular filtration rate, and the results were expressed in mL/min/1.73 m^2^. History of diabetes was defined as fasting plasma glucose level of 126 mg/dL or higher, 2-hour postload plasma glucose level of 200 mg/dL or higher, glycated hemoglobin A_1c_ level of 6.5% or higher, or receiving treatment. Body mass index was calculated as weight in kilograms divided by height in meters squared.

Weekly mean values of physical activity were calculated by multiplying by 7 the self-reported daily engagement in moderate or vigorous physical activity. Physical activity was considered to be at goal if the participant engaged in at least 150 minutes of moderate-intensity or at least 75 minutes of vigorous-intensity aerobic physical activity per week based on national recommendations.^19^ We also tallied the number of coexisting medical conditions at visit 1 (hypertension, diabetes, stroke or TIA, obesity, smoking, and physical activity not at goal) and calculated the total number of vascular risk factors.

### Statistical Analysis

Based on the frequency distribution, we categorized adherence to a Mediterranean diet as follows: low (MDS, 0-4 points), moderate (MDS, 5-6 points), and high (MDS, 7-9 points). Descriptive statistics for each intake category were expressed using weighted means with 95% CIs for continuous variables or total numbers and weighted frequencies for categorical variables. Weighted means and SDs for the cognitive tests were used to generate *z* scores. A global cognition score was created by averaging the mean *z* scores for each neurocognitive test (B-SEVLT Sum, B-SEVLT Recall, word fluency, and DSST).^20^ Overall significance for each cognitive test and Mediterranean diet adherence was calculated with *P* for trend. In pairwise comparisons, the low adherence group was used as the reference category. Clinical and sociodemographic characteristics at visit 1 were compared across the 3 adherence groups by univariate complex sample analysis of variance and χ^2^ tests. The association between MDS and neurocognitive performance at each visit was estimated using complex sample linear regression analysis with least significant difference and adjusting for multiple comparisons.

We constructed linear regression models to estimate the crude and adjusted (per models 1, 2, and 3) cognitive performance for each test. The analytic models included variables known to alter cognition. Model 1 was adjusted by age at the time of the cognitive assessment and sex. Model 2 was adjusted by age, sex, and educational level. Model 3 was adjusted by differences in sociodemographic and clinical characteristics (age, sex, educational level, language preference, history of hypertension, history of stroke or TIA, current or previous smoking status, health insurance, household income, US-born, physical activity, kidney function, body mass index, and number of vascular risk factors, which had *P* < .05) at visit 1. The change in each cognitive score between the 2 visits was calculated as cognitive score at visit 2 minus cognitive score at visit 1, adjusted by the time elapsed between the 2 visits and the cognitive score at visit 1. Mean changes in cognitive score for the different adherence groups were compared by linear regression analysis, with the low adherence group serving as the reference group. The results were adjusted by the 3 models. Differences were considered to be significant at 2-sided *P* < .05. Results were expressed as means and regression coefficients (β estimates) with corresponding 95% CIs.

Statistical analyses were performed using the Complex Samples module in SPSS, version 27 (IBM SPSS), accounting for stratification, clustering, and sampling weights. Taylor series linearization method for variance estimation and correct SE calculations was used.^21^ Data were analyzed from September 2021 to May 2022.

## Results

Of the 6377 participants enrolled in SOL–INCA, 6321 (99.1%) had completed at least 1 dietary recall and were included in the analysis; approximately 90% of participants (n = 5718) had 2 dietary recalls. The cohort had a mean (SE) age of 56.1 (0.18) years at visit 1 and included 4077 women (57.8%) and 2244 men (42.2%). Table 1 depicts consumption of foods for the different components of the MDS. Mediterranean diet adherence weighted frequencies were 35.8% (n = 2112 of 6321) for the low adherence group, 45.4% (n = 2795) for the moderate adherence group, and 18.8% (n = 1414) for the high adherence group. The frequency distribution by MDS category is shown in eTable 1 in the Supplement. The mean MDS was 5.05 (95% CI, 6.98-5.12) points.

Sociodemographic and clinical characteristics by Mediterranean diet adherence group at visit 1 are shown in Table 2. Sex, history of diabetes, and total cholesterol and low-density lipoprotein cholesterol levels were not statistically different between groups. Compared with participants in the low adherence group, adults in the moderate and high adherence groups were less likely to be born in the US (14.0% [n = 328] vs 5.2% [n = 166] and 4.4% [n = 62]), less likely to use English as their preferred language (21.6% [n = 466] vs 9.6% [n = 231] and 7.1% [n = 91]), and less likely to have health insurance (62.9% [n = 1339] vs 51.8% [n = 1405] and 51.7% [n = 685]). In addition, compared with the low adherence group, the high adherence group had a better cardiovascular profile, as evidenced by a lower prevalence of history of hypertension (45.8% [n = 927] vs 38.5% [n = 505]; *P* = .002), history of stroke or TIA (4.5% [n = 78] vs 1.8% [n = 32]; *P* = .001), and current or previous smoking status (24.0% [n = 488] vs 10.8% [n = 147]; *P* < .001); a lower mean number of vascular risk factors (2.2 [95% CI, 2.1-2.2] vs 2.0 [95% CI, 1.9-2.1); *P* < .001) and body mass index (30.3 [95% CI, 29.9-30.6] vs 29.4 [95% CI, 29.1-29.8]; *P* < .001); and better kidney function (estimated glomerular filtration rate, 98.7 [95% CI, 97.2-100.1] vs 103.5 [95% CI, 101.6-105.3] mL/min/1.73 m^2^; *P* < .001).

**Table 2.** Characteristics of Hispanic or Latino Adults at Visit 1 by Mediterranean Diet Adherence

Variable	Low adherence group [n = 2112], No. (weighted %)	Moderate adherence group (n = 2795)^a^		High adherence group (n = 1414)^a^	
		No. (weighted %)	*P* value	No. (weighted %)	*P* value
Sociodemographic factors at visit 1					
Age, mean (95% CI), y	55.7 (55.2-56.3)	56.5 (56.0-57.0)	.04	56.1 (55.4-56.9)	.36
Female sex	1360 (59.1)	1795 (56.9)	.28	922 (57.7)	.54
Male sex	752 (40.9)	1000 (43.1)	.28	492 (42.3)	.54
Household income, $					
<20 000	1013 (47.5)	1297 (46.4)	.61	624 (43.1)	.11
20 000-50 000	721 (32.0)	1057 (35.4)	.08	563 (37.9)	.01
>50 000	197 (9.9)	217 (9.3)	.64	140 (12.6)	.11
Not reported	181 (10.6)	224 (8.8)	.14	87 (6.3)	.003
Educational level					
<High school	770 (35.2)	1137 (35.3)	.98	704 (43.8)	.001
Completed high school	510 (23.3)	595 (21.4)	.25	229 (16.7)	<.001
>High school	830 (41.5)	1053 (43.2)	.39	475 (38.9)	.28
Health insurance	1339 (62.9)	1405 (51.8)	<.001	685 (51.7)	<.001
English as preferred language	466 (21.6)	231 (9.6)	<.001	91 (7.1)	<.001
US-born	328 (14.0)	166 (5.2)	<.001	62 (4.4)	<.001
Clinical characteristics at visit 1					
History of hypertension	927 (45.8)	1052 (41.3)	.02	505 (38.5)	.002
History of diabetes	565 (26.6)	761 (27.9)	.49	427 (29.3)	.26
History of stroke or TIA	78 (4.5)	79 (3.3)	.18	32 (1.8)	.001
Cholesterol, mean (95% CI), mg/dL					
Total	209.4 (206.9-211.8)	211.1 (208.6-213.6)	.29	212.1 (208.2-216.0)	.24
HDL-C	50.5 (49.6-51.4)	49.3 (48.7-50.0)	.04	49.7 (48.6-50.8)	.27
LDL-C	130.1 (127.9-132.3)	132.1 (129.9-134.3)	.19	130.9 (127.2-134.6)	.72
Kidney function, mean (95% CI), mL/min/1.73 m^2^	98.7 (97.2-100.1)	101.5 (100.1-102.9)	.005	103.5 (101.6-105.3)	<.001
Current or previous smoking status	488 (24.0)	458 (17.5)	<.001	147 (10.8)	<.001
Exercise at goal					
Moderate (>150 min/wk)	1133 (52.9)	1556 (55.1)	.27	797 (56.7)	.17
Vigorous (>75 min/wk)	369 (17.1)	533 (19.3)	.18	281 (22.1)	.02
BMI, mean (95% CI)	30.3 (29.9-30.6)	29.7 (29.4-30.0)	.01	29.4 (29.1-29.8)	<.001
Obesity (BMI ≥30)	983 (44.7)	1189 (40.8)	.06	624 (40.2)	.07
No. of vascular risk factors, mean (95% CI)	2.2 (2.1-2.2)	2.1 (2.0-2.1)	<.001	2.0 (1.9-2.1)	<.001

Abbreviations: BMI, body mass index (calculated as weight in kilograms divided by height in meters squared); HDL-C, high-density lipoprotein cholesterol; LDL-C, low-density lipoprotein cholesterol; TIA, transient ischemic attack.

SI conversion factor: To convert HDL-C, LDL-C, and total cholesterol to millimoles per liter, multiply by 0.0259.

^a^ *P* values were calculated using the low adherence group as the reference.

At visit 1, adults in the high adherence group had higher crude (*z* score) B-SEVLT Sum (β = 0.16; 95% CI, 0.06-0.26), B-SEVLT Recall (β = 0.22; 95% CI, 0.12-0.31), and global cognition (β = 0.10; 95% CI, 0.02-.18) scores than adults in the low adherence group. In the fully adjusted model, cognitive scores at visit 1 in the high vs low adherence groups remained higher for B-SEVLT Sum (β = 0.11; 95% CI, 0.02-0.20), B-SEVLT Recall (β = 0.16; 95% CI, 0.07-0.25), and global cognition (β = 0.10; 95% CI, 0.04-0.16) scores (Table 3). At visit 2, adults in the high adherence group had higher crude (*z* score) B-SEVLT Sum (β = 0.22; 95% CI, 0.13-0.31), B-SEVLT Recall (β = 0.24; 95% CI, 0.14-0.34), and global cognition (β = 0.09; 95% CI, 0.02-0.17) scores than those in the low adherence group (Table 4). These differences remained significant after adjustment by age, sex, educational level, and baseline characteristics.

**Table 3.** Association of Mediterranean Diet Adherence With Cognitive Performance at Visit 1

Cognitive test	No. of participants	Mean scores (95% CI)			*P* value for trend	Mean differences, β (95% CI)	
		Low adherence group (n = 2112)	Moderate adherence group (n = 2795)	High adherence group (n = 1414)		Moderate vs low adherence groups	High vs low adherence groups
**Test metrics**							
B-SEVLT Sum							
Crude	6288	22.45 (22.08 to 22.81)	22.74 (22.37 to 23.11)	23.33 (22.90 to 23.76)	.005	0.47 (−0.17 to 0.76)	0.88 (0.35 to 1.42)
Model 1^a^	6288	22.16 (21.82 to 22.51)	22.65 (22.30 to 23.00)	23.15 (22.74 to 23.56)	<.001	0.49 (0.05 to 0.92)	0.98 (0.48 to 1.49)
Model 2^b^	6270	22.01 (21.70 to 22.32)	22.46 (22.13 to 22.79)	23.16 (22.79 to 23.54)	<.001	0.45 (0.06 to 0.83)	1.17 (0.71 to 1.63)
Model 3^c^	5635	23.26 (22.55 to 23.97)	23.38 (22.71 to 24.06)	23.86 (23.13 to 24.59)	.03	0.15 (−0.25 to 0.54)	0.60 (0.11 to 1.10)
B-SEVLT Recall							
Crude	6292	8.02 (7.85 to 8.19)	8.23 (8.07 to 8.40)	8.64 (8.41 to 8.87)	<.001	0.21 (−0.01 to 0.44)	0.62 (0.35 to 0.89)
Model 1^a^	6292	7.88 (7.72 to 8.05)	8.19 (8.04 to 8.35)	8.56 (8.35 to 8.77)	<.001	0.30 (0.09 to 0.52)	0.67 (0.41 to 0.94)
Model 2^b^	6274	7.82 (7.66 to 7.98)	8.11 (7.96 to 8.26)	8.56 (8.36 to 8.77)	<.001	0.29 (0.08 to 0.49)	0.75 (0.51 to 0.98)
Model 3^c^	5639	8.79 (8.38 to 9.19)	8.95 (8.53 to 9.38)	9.28 (8.85 to 9.71)	<.001	0.17 (−0.04 to 0.38)	0.47 (0.21 to 0.72)
Word fluency							
Crude	6217	18.48 (17.99 to 18.96)	18.82 (18.36 to 19.29)	19.03 (18.48 to 19.59)	.29	0.35 (−0.27 to 0.97)	0.56 (−0.17 to 1.29)
Model 1^a^	6217	18.41 (17.94 to 18.89)	18.83 (18.38 to 19.28)	19.01 (18.45 to 19.57)	.22	0.42 (−0.20 to 1.0)	0.59 (−0.14 to 1.32)
Model 2^b^	6200	18.17 (17.72 to 18.63)	18.53 (18.10 to 18.97)	19.08 (18.57 to 19.59)	.03	0.36 (−0.22 to 0.95)	0.89 (0.21 to 1.59)
Model 3^c^	5584	19.86 (18.67 to 21.06)	20.05 (18.92 to 21.18)	20.24 (19.07 to 21.40)	.53	0.17 (−0.41 to 0.75)	0.52 (−0.15 to 1.10)
DSST							
Crude	6177	35.34 (34.41 to 36.28)	34.57 (33.66 to 35.48)	34.45 (33.28 to 35.63)	.33	−0.78 (−1.94 to 0.38)	−0.89 (−2.32 to 0.54)
Model 1^a^	6177	34.96 (34.12 to 35.80)	34.65 (33.76 to 35.53)	34.26 (33.15 to 35.37)	.57	−0.33 (−1.40 to 0.78)	−0.65 (−1.93 to 0.63)
Model 2^b^	6218	34.51 (33.75 to 35.27)	34.03 (33.23 to 34.83)	34.55 (33.61 to 35.48)	.54	−0.50 (−1.50 to 0.49)	0.13 (−0.96 to 1.23)
Model 3^c^	5597	41.99 (40.35 to 43.62)	42.35 (40.69 to 44.01)	42.37 (40.72 to 44.02)	.66	0.31 (−0.58 to 1.20)	0.43 (−0.62 to 1.50)
***z*-Scored metrics**							
B-SEVLT Sum							
Crude	6288	−0.10 (−0.17 to −0.04)	−0.05 (−0.12 to 0.02)	0.06 (−0.02 to 0.13)	.005	0.05 (−0.03 to 0.14)	0.16 (0.06 to 0.26)
Model 1^a^	6288	−0.15 (−0.22 to −0.09)	−0.07 (−0.13 to .0001)	0.02 (−0.05 to 0.10)	<.001	0.09 (0.01 to 0.17)	0.18 (0.09 to 0.27)
Model 2^b^	6270	−0.18 (−0.24 to −0.13)	−0.10 (−0.16 to −0.04)	0.03 (−0.04 to 0.09)	<.001	0.08 (0.01 to 0.15)	0.21 (0.13 to 0.29)
Model 3^c^	5635	0.04 (−0.09 to 0.17)	0.07 (−0.06 to 0.19)	0.15 (0.02 to 0.28)	.03	0.03 (−0.05 to 0.10)	0.11 (0.02 to 0.20)
B-SEVLT Recall							
Crude	6292	−0.14 (−0.20 to −0.08)	−0.07 (−0.13 to −0.01)	0.07 (−0.01 to 0.15)	<.001	0.08 (−0.004 to 0.15)	0.22 (0.12 to 0.31)
Model 1^a^	6292	−0.19 (−0.25 to −0.13)	−0.08 (−0.14 to −0.03)	0.05 (−0.03 to 0.12)	<.001	0.11 (0.03 to 0.18)	0.24 (0.14 to 0.33)
Model 2^b^	6274	−0.21 (−0.27 to −0.16)	−0.11 (−0.16 to −0.06)	0.05 (−0.02 to 0.12)	<.001	0.10 (0.03 to 0.17)	0.26 (0.18 to 0.35)
Model 3^c^	5639	0.13 (−0.02 to 0.27)	0.18 (0.03 to 0.33)	0.30 (0.15 to 0.45)	<.001	0.06 (−0.01 to 0.13)	0.16 (0.07 to 0.25)
Word fluency							
Crude	6217	−0.01 (−0.08 to 0.05)	0.04 (−0.03 to 0.10)	0.06 (−0.01 to 0.14)	.29	0.05 (−0.04 to 0.14)	0.08 (−0.02 to 0.18)
Model 1^a^	6217	−0.02 (−0.09 to 0.04)	0.04 (−0.03 to 0.10)	0.06 (−0.02 to 0.14)	.22	0.06 (−0.03 to 0.14)	0.08 (−0.02 to 0.18)
Model 2^b^	6200	−0.06 (−0.12 to 0.01)	−0.005 (−0.07 to 0.06)	0.07 (0.00004 to 0.14)	.03	0.05 (−0.03 to 0.13)	0.13 (0.03 to 0.22)
Model 3^c^	5584	0.18 (0.01 to 0.35)	0.21 (0.05 to 0.36)	0.23 (0.07 to 0.39)	.53	0.02 (−0.06 to 0.10)	0.07 (−0.02 to 0.17)
DSST							
Crude	6177	0.06 (−0.01 to 0.13)	0.0001 (−0.07 to 0.07)	−0.01 (−0.09 to 0.08)	.33	−0.06 (−0.15 to 0.03)	−0.07 (−0.18 to 0.04)
Model 1^a^	6177	0.03 (−0.03 to 0.10)	0.01 (−0.06 to 0.08)	−0.02 (−0.10 to 0.06)	.57	−0.03 (−0.11 to 0.06)	−0.05 (−0.15 to 0.05)
Model 2^b^	6218	−0.001 (−0.06 to 0.06)	−0.04 (−0.10 to 0.02)	0.002 (−0.07 to 0.07)	.54	−0.04 (−0.11 to 0.04)	0.01 (−0.07 to 0.09)
Model 3^c^	5597	0.56 (0.44 to 0.69)	0.59 (0.47 to 0.72)	0.59 (0.47 to 0.72)	.66	0.02 (−0.04 to 0.09)	0.03 (−0.05 to 0.11)
Global cognition							
Crude	6308	−0.06 (−0.11 to −0.01)	−0.03 (−0.08 to 0.02)	0.04 (−0.02 to 0.10)	.04	0.03 (−0.03 to 0.10)	0.10 (0.02 to 0.18)
Model 1^a^	6308	−0.10 (−0.14 to −0.05)	−0.04 (−0.09 to 0.02)	0.02 (−0.04 to 0.08)	.005	0.06 (0.0001 to 0.12)	0.12 (0.05 to 0.19)
Model 2^b^	6290	−0.12 (−0.16 to −0.08)	−0.07 (−0.12 to −0.02)	0.03 (−0.02 to 0.09)	<.001	0.05 (0.0001 to 0.10)	0.16 (0.09 to 0.22)
Model 3^c^	5653	0.22 (0.12 to 0.32)	0.25 (0.16 to 0.35)	0.31 (0.21 to 0.42)	.006	0.03 (−0.01 to 0.08)	0.10 (0.04 to 0.16)

Abbreviations: B-SEVLT, Brief Spanish-English Verbal Learning Test; DSST, Digit Symbol Substitution Test.

^a^ Adjusted by age and sex.

^b^ Adjusted by age, sex, and educational level.

^c^ Adjusted by age, sex, educational level, language preference, history of hypertension, history of stroke or transient ischemic attack, current or previous smoking status, health insurance, household income, US-born, physical activity, kidney function, body mass index, and number of vascular risk factors.

**Table 4.** Association of Mediterranean Diet Adherence With Cognitive Performance at Visit 2

Cognitive test	No. of participants	Mean scores (95% CI)			*P* value for trend	Mean differences, β (95% CI)	
		Low adherence group (n = 2112)	Moderate adherence group (n = 2795)	High adherence group (n = 1414)		Moderate vs low adherence groups	High vs low adherence groups
**Test metrics**							
B-SEVLT Sum							
Crude	6298	22.20 (21.81 to 22.58)	22.91 (22.59 to 23.23)	23.49 (23.05 to 23.92)	<.001	0.71 (0.26 to 1.17)	1.29 (0.75 to 1.83)
Model 1^a^	6298	21.89 (21.52 to 22.26)	22.82 (22.53 to 23.11)	23.31 (22.90 to 23.72)	<.001	0.93 (0.51 to 1.30)	1.42 (0.92 to 1.92)
Model 2^b^	6280	21.78 (21.42 to 22.13)	22.67 (22.40 to 22.93)	23.40 (22.99 to 23.80)	<.001	0.89 (0.49 to 1.30)	1.62 (1.13 to 2.11)
Model 3^c^	5688	22.72 (21.85 to 23.59)	23.21 (22.43 to 24.00)	23.75 (22.86 to 24.64)	<.001	0.51 (0.13 to 0.90)	1.15 (0.67 to 1.63)
B-SEVLT Recall							
Crude	6286	7.96 (7.76 to 8.16)	8.23 (8.07 to 8.40)	8.67 (8.43 to 8.92)	<.001	0.27 (0.02 to 0.52)	0.72 (0.41 to 1.02)
Model 1^a^	6286	7.82 (7.62 to 8.01)	8.19 (8.04 to 8.35)	8.59 (8.36 to 8.83)	<.001	0.38 (0.14 to 0.61)	0.78 (0.49 to 1.06)
Model 2^b^	6268	7.77 (7.58 to 7.96)	8.14 (7.98 to 8.29)	8.63 (8.40 to 8.86)	<.001	0.36 (0.13 to 0.59)	0.86 (0.58 to 1.14)
Model 3^c^	5679	8.15 (7.74 to 8.55)	8.37 (7.93 to 8.81)	8.76 (8.29 to 9.24)	<.001	0.23 (0.002 to 0.46)	0.62 (0.33 to 0.91)
Word fluency							
Crude	6276	18.20 (17.77 to 18.63)	18.26 (17.85 to 18.67)	18.03 (17.49 to 18.56)	.80	0.06 (−0.51 to 0.62)	−0.18 (−0.85 to 0.49)
Model 1^a^	6276	18.15 (17.73 to 18.57)	18.31 (17.90 to 18.71)	18.03 (17.50 to 18.56)	.70	0.15 (−0.40 to 0.70)	−0.12 (−0.78 to 0.54)
Model 2^b^	6258	18.01 (17.64 to 18.38)	18.10 (17.71 to 18.50)	18.23 (17.75 to 18.72)	.75	0.09 (−0.43 to 0.60)	0.26 (−.33 to 0.84)
Model 3^c^	5669	20.06 (18.83 to 21.29)	20.14 (18.94 to 21.35)	20.00 (18.68 to 21.33)	.89	0.09 (−0.44 to 0.62)	0.06 (−0.53 to 0.65)
DSST							
Crude	6234	33.00 (32.12 to 33.88)	32.01 (31.16 to 32.87)	32.10 (30.93 to 33.28)	.19	−0.99 (−2.10 to 0.13)	−0.89 (−2.26 to 0.48)
Model 1^a^	6234	32.59 (31.81 to 33.37)	32.12 (31.31 to 32.92)	31.96 (30.88 to 33.03)	.53	−0.49 (−1.50 to 0.53)	−0.59 (−1.82 to 0.63)
Model 2^b^	6218	32.19 (31.50 to 32.87)	31.57 (30.86 to 32.29)	32.28 (31.41 to 33.16)	.26	−0.63 (−1.50 to 0.28)	0.18 (−0.83 to 1.19)
Model 3^c^	5638	38.09 (36.44 to 39.74)	38.08 (36.35 to 39.80)	38.32 (36.55 to 40.09)	.86	−0.06 (−0.90 to 0.79)	0.33 (−0.66 to 1.31)
***z*-Scored metrics**							
B-SEVLT Sum							
Crude	6298	−0.15 (−0.22 to (−0.08)	−0.03 (−0.08 to 0.03)	0.07 (0.001 to 0.15)	<.001	0.12 (0.05 to 0.20)	0.22 (0.13 to 0.31)
Model 1^a^	6298	−0.20 (−0.27 to −0.14)	−0.04 (−0.09 to 0.01)	0.04 (−0.03 to 0.11)	<.001	0.16 (0.09 to 0.23)	0.24 (0.16 to 0.33)
Model 2^b^	6280	−0.22 (−0.28 to −0.16)	−0.07 (−0.12 to −0.02)	0.06 (−0.01 to 0.13)	<.001	0.15 (0.08 to 0.22)	0.28 (0.19 to 0.36)
Model 3^c^	5688	−0.06 (−0.21 to 0.09)	0.02 (−0.11 to 0.16)	0.12 (−0.04 to 0.27)	<.001	0.09 (0.02 to 0.16)	0.18 (0.10 to 0.25)
B-SEVLT Recall							
Crude	6286	−0.15 (−0.22 to −0.09)	−0.6 (−0.12 to −0.01)	0.09 (0.001 to 0.17)	<.001	0.09 (0.01 to 0.18)	0.24 (0.14 to 0.34)
Model 1^a^	6286	−0.20 (−0.27 to −0.14)	−0.08 (−0.13 to −0.02)	0.06 (−0.02 to 0.14)	<.001	0.13 (0.05 to 0.20)	0.26 (0.16 to 0.35)
Model 2^b^	6268	−0.22 (−0.28 to −0.15)	−0.10 (−0.15 to −0.04)	0.07 (−0.01 to 0.15)	<.001	0.12 (0.04 to 0.19)	0.29 (0.19 to 0.38)
Model 3^c^	5679	−0.09 (−0.23 to 0.04)	−0.02 (−0.16 to 0.13)	0.12 (−0.04 to 0.27)	<.001	0.08 (0.001 to 0.15)	0.21 (0.11 to 0.30)
Word fluency							
Crude	6276	0.02 (−0.04 to 0.08)	0.03 (−0.03 to 0.08)	−0.01 (−0.08 to 0.07)	.80	0.01 (−0.07 to 0.09)	−0.02 (−0.12 to 0.07)
Model 1^a^	6276	0.01 (−0.05 to 0.07)	0.03 (−0.02 to 0.09)	−0.01 (−0.08 to 0.07)	.70	0.02 (−0.06 to 0.09)	−0.02 (−0.11 to 0.07)
Model 2^b^	6258	−0.01 (−0.06 to 0.04)	0.004 (−0.05 to 0.06)	0.02 (−0.04 to 0.09)	.75	0.01 (−0.06 to 0.08)	0.04 (−0.05 to 0.12)
Model 3^c^	5669	0.27 (0.10 to 0.44)	0.28 (0.12 to 0.45)	0.26 (0.08 to 0.45)	.89	0.01 (−0.06 to 0.08)	0.01 (−0.07 to 0.09)
DSST							
Crude	6234	0.04 (−0.03 to 0.10)	−0.04 (−0.10 to 0.03)	−0.03 (−0.12 to 0.06)	.19	−0.08 (−0.16 to 0.01)	−0.07 (−0.17 to 0.04)
Model 1^a^	6234	0.01 (−0.05 to 0.07)	−0.03 (−0.09 to 0.03)	−0.04 (−0.12 to 0.04)	.53	−0.04 (−0.12 to 0.04)	−0.05 (−0.14 to 0.05)
Model 2^b^	6218	−0.02 (−0.08 to 0.03)	−0.07 (−0.12 to −0.02)	−0.02 (−0.08 to 0.05)	.26	−0.05 (−0.12 to 0.02)	0.01 (−0.06 to 0.09)
Model 3^c^	5638	0.42 (0.30 to 0.55)	0.42 (0.29 to 0.55)	0.44 (0.31 to 0.58)	.86	−0.01 (−0.07 to 0.06)	0.03 (−0.05 to 0.10)
Global cognition							
Crude	6298	−0.07 (−0.12 to −0.01)	−0.03 (−0.07 to 0.02)	0.03 (−0.03 to 0.09)	.05	0.04 (−0.03 to 0.10)	0.09 (0.02 to 0.17)
Model 1^a^	62 989	−0.10 (−0.15 to −0.05)	−0.03 (−0.07 to 0.01)	0.01 (−0.05 to 0.07)	.004	0.07 (0.01 to 0.12)	0.11 (0.04 to 0.18)
Model 2^b^	6280	−0.12 (−0.16 to −0.08)	−0.06 (−0.10 to −0.02)	0.03 (−0.02 to 0.08)	<.001	0.06 (0.01 to 0.11)	0.16 (0.09 to 0.21)
Model 3^c^	5688	0.13 (0.02 to 0.24)	0.18 (0.07 to 0.28)	0.23 (0.11 to 0.35)	.003	0.04 (−0.01 to 0.09)	0.11 (0.05 to 0.16)

Abbreviations: B-SEVLT, Brief Spanish-English Verbal Learning Test; DSST, Digit Symbol Substitution Test.

^a^ Adjusted by age and sex.

^b^ Adjusted by age, sex, and educational level.

^c^ Adjusted by age, sex, educational level, language preference, history of hypertension, history of stroke or transient ischemic attack, current or previous smoking status, health insurance, household income, US-born, physical activity, kidney function, body mass index, and number of vascular risk factors.

Table 5 depicts the association between Mediterranean diet adherence group and cognitive change between visits (mean elapsed time of 7 years). The change in cognitive performance varied by Mediterranean diet adherence. In the model adjusted for age, sex, and educational level (model 2), the decreases in B-SEVLT Sum mean *z* scores were slower in the moderate vs low adherence groups (β = 0.11; 95% CI, 0.05-0.17) and in the high vs low adherence groups (β = 0.18; 95% CI, 0.10-0.25). Similarly, declines in the mean B-SEVLT Recall *z* scores were slower in the moderate vs low adherence groups (β = 0.08; 95% CI, 0.01-0.15) compared with the high vs low adherence groups (β = 0.17; 95% CI, 0.09-0.26). These differences were attenuated after adding covariates in the model. In the fully adjusted model (model 3), high adherence to the Mediterranean diet remained associated with a slower decline in the B-SEVLT Sum (β = 0.12; 95% CI, 0.05-0.20) and B-SEVLT Recall (β = 0.14; 95% CI, 0.05-0.23) scores, compared with low adherence. The changes in word fluency, DSST score, and global cognition score were not associated with adherence to the Mediterranean diet.

**Table 5.** Cognitive Performance Change Between Visits 1 and 2 by Mediterranean Diet Adherence

Cognitive test	No. of participants	Mean scores (95% CI)			*P* value for trend	Mean differences, β (95% CI)	
		Low adherence group (n = 2112)	Moderate adherence group (n = 2795)	High adherence group (n = 1414)		Moderate vs low adherence groups	High vs low adherence groups
**Test metrics**							
B-SEVLT Sum							
Crude	6266	−0.33 (−0.62 to −0.03)	0.14 (−0.12 to 0.40)	0.42 (0.05 to 0.79)	.002	0.47 (0.12 to 0.81)	0.74 (0.28 to 1.19)
Model 1^a^	6266	−0.51 (−0.81 to −0.21)	0.12 (−0.13 to 0.36)	0.37 (0.01 to 0.74)	<.001	0.63 (0.29 to 0.96)	0.89 (0.42 to 1.31)
Model 2^b^	6248	−0.57 (−0.87 to −0.27)	0.06 (−0.18 to 0.30)	0.48 (0.10 to 0.85)	<.001	0.63 (0.29 to 0.97)	1.02 (0.06 to 1.47)
Model 3^c^	5628	−0.15 (−1.01 to 0.72)	0.25 (−0.56 to 1.07)	0.59 (−0.32 to 1.49)	.003	0.41 (0.07 to 0.74)	0.72 (0.29 to 1.14)
B-SEVLT Recall							
Crude	6258	−0.15 (−0.32 to 0.02)	−0.01 (−0.15 to 0.13)	0.23 (0.02 to 0.44)	.02	0.14 (−0.08 to 0.36)	0.37 (0.10 to 0.64)
Model 1^a^	6258	−0.25 (−0.41 to −0.08)	−0.02 (−0.16 to 0.11)	0.21 (0.01 to 0.42)	.002	0.23 (0.02 to 0.43)	0.45 (0.19 to 0.72)
Model 2^b^	6240	−0.27 (−0.44 to −0.10)	−0.04 (−0.18 to 0.09)	0.26 (0.05 to 0.46)	<.001	0.23 (0.02 to 0.44)	0.52 (0.26 to 0.79)
Model 3^c^	5623	−0.27 (−0.63 to 0.09)	−0.12 (−0.52 to 0.27)	0.14 (−0.30 to 0.57)	.01	0.15 (−0.06 to 0.37)	0.42 (0.15 to 0.70)
Word fluency							
Crude	6183	−0.17 (−0.53 to 0.19)	−0.40 (−0.65 to −0.16)	−0.81 (−0.12 to −0.45)	.04	−0.24 (−0.67 to 0.20)	−0.64 (−1.14 to −0.13)
Model 1^a^	6183	−0.18 (−0.54 to 0.17)	−0.35 (−0.60 to −0.11)	−0.79 (−1.14 to −0.43)	.04	−0.18 (−0.61 to 0.25)	−0.59 (−1.08 to −0.09)
Model 2^b^	6166	−0.23 (−0.56 to 0.11)	−0.39 (−0.65 to −0.14)	−0.67 (−1.03 to −0.31)	.18	−0.18 (−0.60 to 0.24)	−0.39 (−0.89 to 0.10)
Model 3^c^	5565	0.57 (−0.29 to 1.43)	0.49 (−0.32 to 1.31)	0.18 (−0.73 to 1.10)	.23	−0.07 (−0.49 to 0.36)	−0.36 (−0.86 to 0.15)
DSST							
Crude	6093	−2.01 (−2.49 to −1.53)	−2.36 (−2.76 to −1.96)	−2.23 (−2.75 to −1.72)	.54	−0.36 (−0.99 to 0.27)	−0.22 (−0.87 to 0.44)
Model 1^a^	6093	−2.09 (−2.57 to −1.62)	−2.32 (−2.70 to −1.94)	−2.30 (−2.78 to −1.82)	.73	−0.24 (−0.84 to 0.38)	−0.19 (−0.84 to 0.45)
Model 2^b^	6093	−2.2 0 (−2.67 to −1.74)	−2.48 (to 2.87 to −2.10)	−2.28 (−2.77 to −1.80)	.64	−0.29 (to 0.88 to 0.31)	−0.07 (−0.70 to 0.56)
Model 3^c^	5508	−1.76 (−2.94 to −0.58)	−2.10 (−3.30 to −.85)	−1.91 (−3.14 to −0.69)	.61	−0.32 (−0.94 to 0.30)	−0.13 (−0.76 to 0.51)
***z*-Scored metrics**							
B-SEVLT Sum							
Crude	6266	−0.06 (−0.11 to −0.01)	0.02 (−0.03 to 0.06)	0.07 (0.001 to 0.13)	.002	0.08 (0.02 to 0.14)	0.13 (0.05 to 0.20)
Model 1^a^	6266	−0.10 (−0.15 to −0.04)	0.01 (−0.03 to 0.06)	0.06 (−0.01 to 0.12)	<.001	0.11 (0.05 to 0.17)	0.15 (0.07 to 0.23)
Model 2^b^	6248	−0.11 (−0.16 to −0.05)	0.003 (−0.04 to 0.04)	0.07 (0.01 to 0.14)	<.001	0.11 (0.05 to 0.17)	0.18 (0.10 to 0.25)
Model 3^c^	5628	−0.03 (−0.18 to 0.12)	0.04 (−0.10 to 0.18)	0.09 (−0.06 to 0.25)	.003	0.07 (0.01 to 0.13)	0.12 (0.05 to 0.20)
B-SEVLT Recall							
Crude	6258	−0.04 (−0.10 to 0.01)	0.002 (−0.04 to 0.05)	0.08 (0.01 to 0.15)	.02	0.05 (−0.03 to 0.12)	0.12 (0.03 to 0.21)
Model 1^a^	6258	−0.08 (−0.13 to −0.02)	−0.002 (−0.05 to 0.04)	0.08 (0.01 to 0.15)	.002	0.08 (0.01 to 0.15)	0.15 (0.06 to 0.24)
Model 2^b^	6240	−0.09 (−0.14 to −0.03)	−0.01 (−0.05 to 0.04)	0.09 (0.02 to 0.16)	<.001	0.08 (0.01 to 0.15)	0.17 (0.09 to 0.26)
Model 3^c^	5623	−0.08 (−0.21 to 0.04)	−0.04 (−0.17 to 0.10)	0.05 (−0.09 to 0.20)	.01	0.05 (−0.02 to 0.12)	0.14 (0.05 to 0.23)
Word fluency							
Crude	6183	0.04 (−0.02 to 0.05)	0.01 (−0.02 to 0.05)	−0.04 (−0.09 to 0.01)	.04	−0.03 (−0.09 to 0.03)	−0.09 (−0.16 to −0.02)
Model 1^a^	6183	0.04 (−0.01 to 0.09)	0.02 (−0.01 to 0.05)	−0.04 (−0.09 to 0.01)	.04	−0.02 (−0.08 to 0.03)	−0.08 (−0.15 to −0.01)
Model 2^b^	6166	0.04 (−0.01 to 0.08)	0.01 (−0.02 to 0.05)	−0.02 (−0.07 to 0.02)	.18	0.03 (−0.08 to 0.03)	−0.05 (−0.12 to 0.01)
Model 3^c^	5565	0.15 (0.03 to 0.26)	0.14 (0.02 to 0.25)	0.09 (−0.03 to 0.22)	.23	−0.01 (−0.07 to 0.05)	−0.05 (−0.12 to 0.02)
DSST							
Crude	6093	0.001 (−0.04 to 0.04)	−0.03 (−0.06 to 0.01)	−0.02 (−0.06 to 0.02)	.54	−0.03 (−0.08 to 0.02)	−0.02 (−0.07 to 0.03)
Model 1^a^	6093	−0.01 (−0.04 to 0.03)	−0.02 (−0.05 to 0.01)	−0.02 (−0.06 to 0.02)	.73	−0.02 (−0.06 to 0.03)	−0.02 (−0.06 to 0.03)
Model 2^b^	6093	−0.01 (−0.05 to 0.02)	−0.03 (−0.06 to −0.01)	−0.02 (−0.06 to 0.02)	.64	−0.02 (−0.07 to 0.02)	−0.01 (−0.05 to 0.04)
Model 3^c^	5508	0.02 (−0.07 to 0.11)	−0.004 (−0.10 to 0.09)	0.01 (−0.08 to 0.10)	.61	−0.02 (−0.07 to 0.02)	−0.01 (−0.06 to 0.04)
Global cognition							
Crude	6285	−0.01 (−0.04 to 0.02)	−0.002 (−0.03 to 0.02)	0.002 (−0.04 to 0.04)	.89	0.01 (−0.03 to 0.05)	0.01 (−0.04 to 0.06)
Model 1^a^	6285	−0.02 (−0.06 to 0.01)	0.001 (−0.02 to 0.03)	0.002 (−0.04 to 0.04)	.42	0.02 (−0.02 to 0.06)	0.03 (−0.02 to 0.07)
Model 2^b^	6267	−0.03 (−0.06 to 0.002)	−0.005 (−0.03 to 0.02)	0.02 (−0.02 to 0.06)	.13	0.03 (−0.01 to 0.06)	0.05 (0.001 to 0.09)
Model 3^c^	5645	0.01 (−0.08 to 0.10)	0.03 (−0.06 to 0.12)	0.05 (−0.05 to 0.15)	.32	0.02 (−0.02 to 0.06)	0.04 (−0.01 to 0.09)

Abbreviations: B-SEVLT, Brief Spanish-English Verbal Learning Test; DSST, Digit Symbol Substitution Test.

^a^ Adjusted by age and sex.

^b^ Adjusted by age, sex, and educational level.

^c^ Adjusted by age, sex, educational level, language preference, history of hypertension, history of stroke or transient ischemic attack, current or previous smoking status, health insurance, household income, US-born, physical activity, kidney function, body mass index, and number of vascular risk factors.

Compared with the MDS, the modified MDS yielded higher scores (eTable 1 in the Supplement). eTable 2 in the Supplement provides the characteristics at visit 1 by modified MDS adherence groups. Use of the modified MDS had a minimal association with the point estimates and did not alter the final results (eTables 3-5 in the Supplement).

## Discussion

In this cohort of diverse Hispanic or Latino adults, higher adherence to a Mediterranean diet was associated with higher global cognition scores and decreased 7-year learning and memory decline. These findings suggest that diet modification may offer another means of reducing the risk for cognitive impairment. A Mediterranean diet that is culturally tailored for a specific Hispanic or Latino heritage could offer enhanced Mediterranean diet–type benefits.

An association between Mediterranean diet adherence and cognitive performance has been shown in multiple studies. In a meta-analysis of 6 different cohorts, participants in the higher Mediterranean diet tertile had a 33% reduction in the risk of mild cognitive impairment or Alzheimer disease compared with those in the lowest tertiles.^22^ In another meta-analysis including 15 cohort studies and 2 randomized clinical trials, the Mediterranean diet was associated with different cognitive outcomes, such as memory and global cognition.^23^ Because the advantage of the Mediterranean diet for cognition has been observed in individuals with different backgrounds, the benefit was suggested as transferable across populations.^24^ However, the few studies of the implications of the Mediterranean diet for cognitive decline among Hispanic or Latino participants have reported conflicting results. In the cross-sectional sample of the Boston Puerto Rican Health Study (1269 middle-aged and older adults), each point increase in the MDS was associated with a 0.14-point increase in global cognition, as defined by the Mini-Mental State Examination score.^18^ A subsequent study that used longitudinal data from the same cohort (n = 913) reported that higher Mediterranean diet adherence was associated with a 2-year change in memory. However, the benefits on global function and Mini-Mental State Examination scores were observed only in adults with type 2 diabetes, but not in adults without diabetes.^25^ This finding suggests that Mediterranean diet adherence may be protective against age-dependent cognitive decline in a subset of Hispanic or Latino adults. In comparison, a study of the 2011 to 2014 National Health and Nutrition Examination Survey involving 2435 participants found an association between Mediterranean diet and increased cognition among non-Hispanic White adults but not among Hispanic or Latino adults.^26^

Results of the present study support that increased adherence to the Mediterranean diet is associated with enhanced cognition and decreased age-dependent cognitive decline in middle-aged and older Hispanic or Latino adults. These associations were largely derived from the results of the neurocognitive tests that assessed episodic learning and memory (B-SEVLT Sum and B-SEVLT Recall). In a cross-sectional study using visit 1 data of the HCHS/SOL, a healthier diet quality, defined by the Alternate Healthy Eating Index, was associated with better global cognition.^27^ However, similar to the present study, that study observed no associations between healthy diet and word fluency or DSST score.^27^ It is possible that diet is particularly beneficial for neuroanatomical areas that are associated with memory. The neurocircuitry involved in episodic learning and memory relies on the extensive connections among the medial temporal lobe, hippocampus, and frontal cortex.^28^ Mechanistically, the Mediterranean diet was associated with larger temporal lobe and hippocampal volumes.^11^ Furthermore, MDS was associated with preserved brain volume in mediotemporal regions and decreased β-amyloid and phosphorylated tau pathology.^12^ Based on these observations, it was hypothesized that a Mediterranean diet may protect the integrity of brain areas involved in memory and learning from pathologic events, such as amyloid deposition and tau phosphorylation.^11,12^ Alternatively, it has been proposed that the Mediterranean diet is associated with lower risk of dementia because it reduces the prevalence of vascular comorbid conditions that affect the normal functioning of the neurovascular unit.^29^ In the present study, we observed a lower frequency of risk factors associated with cerebrovascular diseases and neurovascular uncoupling in participants in the high adherence group. However, the results remained statistically significant after adjustment for these variables.

Several baseline characteristics, including educational level, English as the preferred language, birth in the US, and health insurance, suggest an inverse association between acculturation and Mediterranean diet adherence. These observations are in agreement with previous studies that found the acculturative process of Hispanic or Latino populations to US society was associated with the adoption of less healthy dietary habits, which can affect the development of chronic medical conditions.^30,31^ Results of the present study remained significant after adjustment for individual sociodemographic variables that were significantly different between groups. However, it is possible that the interplay of social determinants of health, including education, income, and access to health insurance, is a factor in the association between diet quality and cognitive function. We are currently investigating the modulatory implication of social determinants of health for the association between Mediterranean diet adherence and cognitive decline, and we plan to publish these results.

### Limitations

This study has several limitations. First, several covariates were self-reported with the possibility of recall bias. Individuals with low adherence to the Mediterranean diet were more likely to report being born in the US and a preference for using the English language, which were factors in the adoption of unhealthy nutritional behaviors in some, but not all, Hispanic or Latino adults. Although we adjusted for these factors at visit 1, longer time in the study may have hastened the adoption of new dietary and lifestyle habits that may have altered the association between Mediterranean diet that was measured only at baseline and cognitive performance 7 years later.^32^ Thus, long-term diet-measurement methods and extended follow-up may be necessary to identify temporal variations in dietary patterns and their associations with the development of chronic diseases.

Second, we used the original MDS,^8^ and the calculation of this score was based on the consumption of beneficial and detrimental foods above or below sex-specific medians. Although easy to reproduce, the original MDS assumed an outcome that was based on consumption thresholds, which may not be applicable to the diet of specific populations. For example, consumption of refined grains and potatoes is high in some Hispanic or Latino groups.^17^ Thus, a modified version of the MDS was developed for these populations.^16^ In a sensitivity analysis, replacing the MDS with the modified MDS did not change the final results.

Third, we used only 4 tests (B-SEVLT Sum, B-SEVLT Recall, word fluency, and DSST) to assess cognitive performance. We cannot exclude the possibility that including other neurocognitive tests could yield different results. Despite these limitations, to our knowledge, this study analyzed the largest and most diverse cohort of middle-aged and older Hispanic or Latino individuals to understand the association of Mediterranean diet with cognition.

## Conclusions

In this large, longitudinal, population-based cohort study of diverse Hispanic or Latino adults, we found that a higher level of Mediterranean diet adherence was associated with better cognitive function, specifically episodic learning and memory, and with decreased 7-year memory decline, which are 2 cognitive domains related to Alzheimer disease. A culturally tailored Mediterranean diet may reduce the risk of cognitive decline and Alzheimer disease among Hispanic or Latino adults. Future studies should examine whether the results prove similar across different racial and ethnic heritage.

## Notes

Supplement. **eTable 1.** Frequency of Hispanic/Latino Adults for Each Intake Category at Visit 1 by the Mediterranean Diet Score and the Modified Mediterranean Diet Score (N=6321) **eTable 2.** Characteristics of Hispanic or Latino Adults at Visit 1 by Modified Mediterranean Diet Adherence **eTable 3.** Association of Modified Mediterranean Diet Score With Cognitive Performance at Visit 1 **eTable 4.** Association of Modified Mediterranean Diet Score With Cognitive Performance at Visit 2 **eTable 5.** Cognitive Performance Change Between Visits 1 and 2 by Modified Mediterranean Diet Adherence
