# Supplementary material for: Association of Mediterranean Diet With Cognitive Decline Among Diverse Hispanic or Latino Adults From the Hispanic Community Health Study/Study of Latinos
Source: JAMA Netw Open. 2022 Jul 14;5(7):e2221982. doi: 10.1001/jamanetworkopen.2022.21982 (PMC9284337; doi:10.1001/jamanetworkopen.2022.21982)
Supplement: Supplement. — eTable 1. Frequency of Hispanic/Latino Adults for Each Intake Category at Visit 1 by the Mediterranean Diet Score and the Modified Mediterranean Diet Score (N=6321) eTable 2. Characteristics of Hispanic or Latino Adults at Visit 1 by Modified Mediterranean Diet Adherence eTable 3. Association of Modified Mediterranean Diet Score With Cognitive Performance at Visit 1 eTable 4. Association of Modified Mediterranean Diet Score With Cognitive Performance at Visit 2 eTable 5. Cognitive Performance Change Between Visits 1 and 2 by Modified Mediterranean Diet Adherence [file jamanetwopen-e2221982-s001.pdf]

Supplemental Online Content

Moustafa B, Trifan G, Isasi CR, et al. Association of Mediterranean diet with cognitive decline among diverse Hispanic or Latino adults from the Hispanic Community Health Study/Study of Latinos. *JAMA Netw Open*. 2022;5(7):e2221982. doi:10.1001/jamanetworkopen.2022.21982

eTable 1. Frequency of Hispanic/Latino Adults for Each Intake Category at Visit 1 Using the Mediterranean Diet Score and the Modified Mediterranean Diet Score (N=6321)

eTable 2. Characteristics of Hispanic or Latino Adults at Visit 1 by Modified Mediterranean Diet Adherence

eTable 3. Association of Modified Mediterranean Diet Score With Cognitive Performance at Visit 1

eTable 4. Association of Modified Mediterranean Diet Score With Cognitive Performance at Visit 2

eTable 5. Cognitive Performance Change Between Visits 1 and 2 by Modified Mediterranean Diet Adherence

This supplemental material has been provided by the authors to give readers additional information about their work.

© 2022 Moustafa B et al. *JAMA Network Open.*

eTable 1. Frequency of Hispanic/Latino Adults for Each Intake Category at Visit 1 Using the Mediterranean Diet Score and the Modified Mediterranean Diet Score (N=6321)

	**Mediterranean Diet Score**	**Modified Mediterranean Diet Score**
**Score**	**Weighted Frequency (95% CI)**	**Weighted Frequency (95% CI)**
0	0.04 (0.01-0.2)	0.05 (0.01-0.2)
1	1.0 (0.7-1.4)	1.0 (0.8-1.3)
2	5.4 (4.4-6.5)	5.9 (4.9-7.1)
3	11.7 (10.6-12.8)	11.9 (10.8-13.2)
4	17.7 (16.4-19.2)	16.2 (14.8-17.6)
5	23.8 (22.1-25.5)	21.9 (20.4-23.6)
6	21.7 (20.3-23.1)	22.2 (20.6-24.0)
7	12.8 (11.6-14.0)	13.4 (12.2-14.8)
8	5.0 (4.3-5.8)	5.9 (5.1-6.9)
9	1.0 (0.8-1.3)	1.4 (1.1-1.8)

© 2022 Moustafa B et al. *JAMA Network Open.*

eTable 2. Characteristics of Hispanic or Latino Adults at Visit 1 by Modified Mediterranean Diet Adherence

Variable	Low adherence group (n = 2020) No. (weighted %)	Moderate adherence group (n = 2741)[a]		Moderate adherence group (n = 1560)[a]	
		No. (weighted %)	P Value	No. (weighted %)	P value
**Sociodemographic factors at Visit 1**					
Age (years)	55.8 (55.3-56.4)	56.3 (55.7-56.8)	.28	56.5 (55.8-57.1)	P=.12
Sex					
Female	1312 (59.8)	1754 (56.3.)	.10	1011 (57.8)	.35
Male	708 (40.2)	987 (43.7)		549 (42.2)	
Household income					
<$20,000	978 (48.2)	1278 (46.8)	.52	678 (41.5)	.01
$20,000-50,000	682 (31.3)	1029 (35.5)	.04	630 (38.4)	.002
>$50,000	183 (9.8)	210 (8.7)	.42	161 (13.9)	.01
Not reported	177 (10.8)	224 (8.9)	.14	91 (6.2)	.003
Educational level					
Less than high school	725 (34.8)	1108 (35.3)	.83	778 (43.6)	.001
Completed high school	505 (23.7)	580 (21.3)	.15	249 (16.8)	.001
More than high school	789 (41.4)	1043 (43.3)	.36	526 (39.0)	.32
Health insurance	1275 (62.6)	1384 (52.9)	.001	770 (53.0)	.001
English as preferred language	452 (21.1)	236 (10.2)	.001	100 (7.3)	.001
US born	313 (13.4)	170 (5.5)	.001	73 (5.0)	.001
**Clinical characteristics at Visit 1**					
History of hypertension	891 (46.2)	1028 (41.2)	.02	565 (38.7)	.001
History of diabetes	533 (26.5)	759 (28.2)	.37	461 (28.5)	.37
History of stroke or TIA	74 (4.4)	78 (3.4)	.29	37 (2.0)	.002
Cholesterol (mg/dL)					
Total	210.2 (207.5-212.9)	210.2 (207.7-212.7)	.99	212.5 (208.5-216.5)	.36
HDL-C	50.6 (49.6-51.5)	49.2 (48.5-49.8)	.02	49.9 (48.9-51.0)	.38
LDL-C	130.9 (128.6-133.3)	130.9 (128.6-133.2)	.98	132.0 (128.2-135.8)	.65
Kidney function (ml/min/1.73m^2)	98.3 (96.8-99.7)	101.7 (100.2-103.2)	.002	103.5 (101.8-105.2)	.001
Current or previous smoking	496 (25.6)	441 (16.8)	.001	156 (10.4)	.001
Exercise at goal					
Moderate (> 150 min/week)	1074 (52.6)	1526 (54.8)	30	886 (57.2)	.08

© 2022 Moustafa B et al. *JAMA Network Open.*

Vigorous (> 75 min/week)	347 (16.8)	517 (19.4)	.11	319 (21.9)	.009
Body mass index (95% CI)	30.2 (29.8-30.6)	29.8 (29.4-30.1)	.08	29.4 (29.1-29.8)	.002
Obesity (BMI ≥30)	930 (44.0)	1186 (41.6)	.25	680 (39.9)	.08
Number of Vascular Risk Factors (95% CI)	2.2 (2.1-2.2)	2.1 (2.0-2.1)	.03	2.0 (1.9-2.1)	.001

Abbreviations: BMI, body mass index (calculated as weight in kilograms divided by height in meters squared); HDL-C, high-density lipoprotein cholesterol; LDL-C, low density lipoprotein cholesterol; TIA, transient ischemic attack; SI conversion factor: To convert HDL-C, LDL-C, and total cholesterol to millimoles per liter, multiply by 0.0259.

[a]P values were calculated using the low adherence group as the reference.

© 2022 Moustafa B et al. *JAMA Network Open.*

eTable 3. Association of Modified Mediterranean Diet Score With Cognitive Performance at Visit 1

Cognitive Test	Mean scores (95% CI)			P value for trend	Mean differences, β (95% CI)	
	Low adherence group (n = 2020)	Moderate adherence group (n = 2741)	High adherence group (n = 1560)		Moderate vs. low adherence groups	High vs. low adherence groups
**Test Metrics**						
**B-SEVLT-Sum**						
Crude (n=6288)	22.32 (21.93-22.70)	22.68 (22.34-23.03)	23.61 (23.15-24.07)	<.001	0.37 (-0.11-0.85)	1.29 (0.73-1.86)
Model 1[a] (n=6288)	22.04 (21.68-22.40)	22.56 (22.24-22.88)	23.49 (23.03-23.95)	<.001	0.52 (0.08-0.96)	1.44 (0.90-1.99)
Model 2[b] (n=6270)	21.88 (21.56-22.20)	22.37 (22.07-22.68)	23.50 (23.08-23.91)	<.001	0.49 (0.10-0.89)	1.64 (1.13-2.14)
Model 3[c] (n=5635)	23.14 (22.43-23.85)	23.31 (22.64-23.98)	24.17 (23.42-24.91)	<.001	0.17 (-0.22-0.57)	1.08 (0.54-1.62)
**B-SEVLT-Recall**						
Crude (n=6292)	7.99 (7.82-8.16)	8.17 (8.02-8.33)	8.77 (8.54-8.99)	<.001	0.18 (-0.04-0.41)	0.78 (0.50-1.05)
Model 1[a] (n=6292)	7.86 (7.69-8.03)	8.12 (7.97-8.26)	8.71 (8.49-8.93)	<.001	0.26 (0.04-0.47)	0.85 (0.59-1.12)
Model 2[b] (n=6274)	7.79 (7.63-7.95)	8.04 (7.89-8.18)	8.72 (8.51-8.93)	<.001	0.24 (0.04-0.45)	0.94 (0.69-1.18)
Model 3[c] (n=5639)	8.77 (8.36-9.17)	8.87 (8.45-9.30)	9.41 (8.98-9.84)	<.001	0.11 (-0.10-0.31)	0.63 (0.37-0.89)
**Word fluency**						
Crude (n=6217)	18.41 (17.91-18.91)	18.67 (18.20-19.97)	19.43 (18.89-19.97)	.02	0.25 (-0.40-0.90)	1.02 (0.31-1.73)
Model 1[a] (n=6217)	18.36 (17.87-18.84)	18.66 (18.20-19.11)	19.44 (18.89-19.98)	.01	0.31 (-0.33-0.94)	1.06 (0.35-1.77)
Model 2[b] (n=6200)	18.11 (17.65-18.57)	18.36 (17.92-18.79)	19.51 (18.99-20.03)	<.001	0.25 (-0.35-0.85)	1.37 (0.67-2.06)
Model 3[c] (n=5584)	19.82 (18.63-21.01)	19.88 (18.76-21.00)	20.66 (19.47-21.86)	.02	0.05 (-0.54-0.64)	0.97 (0.30-1.64)
**DSST**						
Crude (n=6177)	35.33 (34.34-36.33)	34.43 (33.57-35.29)	34.81 (33.71-35.90)	.32	-0.90 (-2.1-0.27)	-0.53 (-1.88-0.82)
Model 1[a] (n=6177)	35.00 (34.12-35.89)	34.35 (33.50-35.21)	34.86 (33.81-35.90)	.48	-0.66 (-1.8-0.45)	-0.08(-1.32-1.16)
Model 2[b] (n=6218)	34.52 (33.73-35.31)	33.74 (32.96-34.52)	35.13 (34.24-36.02)	.03	-0.79 (-1.79-0.20)	0.70 (-0.37-1.77)
Model 3[c] (n=5597)	42.09 (40.46-43.73)	42.05 (40.38-43.71)	42.79 (41.16-44.43)	.29	-0.12 (-1.00-0.77)	0.79 (-0.28-1.85)

© 2022 Moustafa B et al. *JAMA Network Open.*

**Z-scored Metrics**						
**B-SEVLT-Sum**						
Crude (n=6288)	-0.13 (-0.20-(-)0.06)	-0.06 (-0.12 -0.0001)	0.11 (0.02-0.19)	<.001	0.07 (-0.02-0.15)	0.23 (0.13-0.34)
Model 1[a] (n=6288)	-0.18 (-0.24-(-)0.11)	-0.08 (-0.14-(-)0.02)	0.09 (0.003-0.17)	<.001	0.09 (0.02-0.17)	0.26 (0.16-0.36)
Model 2[b] (n=6270)	-0.21 (-0.26-(-)0.15)	-0.12 (-0.17-(-)0.06)	0.09 (0.01-0.16)	<.001	0.09 (0.02-0.16)	0.29 (0.20-0.38)
Model 3[c] (n=5635)	0.02 (-0.11-0.15)	0.05 (-0.07-0.17)	0.21 (0.07-0.34)	<.001	0.04 (-0.03-0.11)	0.23 (0.12-0.33)
**B-SEVLT-Recall**						
Crude (n=6292)	-0.15 (-0.21-(-)0.09)	-0.09 (-0.14-(-)0.03)	0.12 (0.04-0.20)	<.001	0.06 (-0.01-0.14)	0.27 (0.17-0.34)
Model 1[a] (n=6292)	-0.20 (-0.26-(-)0.14)	-0.11 (-0.16-(-)0.06)	0.10 (0.02-0.18)	<.001	0.09 (0.01-0.17)	0.29 (0.20-0.39)
Model 2[b] (n=6274)	-0.22 (-0.28-(-)0.17)	-0.14 (-0.19-(-)0.09)	0.10 (0.03-0.17)	<.001	0.08 (0.01-0.15)	0.33 (0.24-0.41)
Model 3[c] (n=5639)	0.12 (-0.02-0.26)	0.16 (0.01-0.31)	0.34 (0.19-0.50)	<.001	0.05 (-0.03-0.12)	0.23 (0.14-0.33)
**Word fluency**						
Crude (n=6217)	-0.02 (-0.09-0.05)	0.01 (-0.05-0.08)	0.12 (0.04-0.20)	.02	0.04 (-0.05-0.13)	0.14 (0.04-0.24)
Model 1[a] (n=6217)	-0.03 (-0.10-0.04)	0.01 (-0.05-0.08)	0.12 (0.04-0.20)	.01	0.04 (-0.05-0.13)	0.15 (0.05-0.25)
Model 2[b] (n=6200)	-0.06 (-0.13-(-)0.002)	-0.03 (-0.09-0.03)	0.13 (0.06-0.20)	<.001	0.04 (-0.05-0.12)	0.19 (0.09-0.29)
Model 3[c] (n=5584)	0.17 (0.01-0.34)	0.18 (0.03-0.34)	0.29 (0.13-0.46)	.02	0.02 (-0.06-0.11)	0.15 (0.05-0.25)
**DSST**						
Crude (n=6177)	0.06 (-0.01-0.14)	-0.01 (-0.07-0.06)	0.02 (-0.06-0.10)	.32	-0.07 (-0.16-0.02)	-0.04 (-0.14-0.06)
Model 1[a] (n=6177)	0.04 (-0.03-0.10)	-0.01 (-0.08-0.05)	0.03 (-0.05-0.10)	.48	-0.05 (-0.13-0.03)	-0.01 (-0.10-0.09)
Model 2[b] (n=6218)	-0.00003 (-0.06-0.06)	-0.06 (-0.12-(-)0.0003)	0.05 (-0.02-0.11)	.03	-0.06 (-0.14-0.02)	0.05 (-0.03-0.13)
Model 3[c] (n=5597)	0.57 (0.45-0.70)	0.57 (0.44-0.69)	0.63 (0.50-0.75)	.29	0.001 (-0.07-0.07)	0.07 (-0.02-0.15)
**Global Cognition**						
Crude (n=6308)	-0.07 (-0.13-(-)0.02)	-0.05 (-0.10-0.0004)	0.09 (0.03-0.15)	<.001	0.02 (-0.04-0.09)	0.14 (0.06-0.21)
Model 1[a] (n=6308)	-0.11 (-0.16-(-)0.05)	-0.06 (-0.11-(-)0.01)	0.08 (0.02-0.14)	<.001	0.05 (-0.01-0.11)	0.16 (0.09-0.24)
Model 2 [b] (n=6290)	-0.13 (-0.18-(-)0.09)	-0.09 (-0.14-(-)0.05)	0.09 (0.04-0.14)	<.001	0.03 (-0.02-0.09)	0.20 (0.140.26)
Model 3 [c] (n=5653)	0.21 (0.12-0.31)	0.23 (0.14-0.33)	0.36 (0.26-0.46)	<.001	0.03 (-0.02-0.08)	0.16 (0.09-0.23)

Abbreviations: B-SEVLT, Brief -Spanish- English Verbal Learning Test; DSST, Digit Symbol Substitution Test.

[a]Adjusted by age and sex.

[b]Adjusted by age, sex, and educational level.

[c]Adjusted by age, sex, educational level, language preference, history of hypertension, history of stroke or transient ischemic attack, current or previous smoking status, health insurance, household income, US born, physical activity, kidney function, body mass index, and number of vascular risk factors.

© 2022 Moustafa B et al. *JAMA Network Open.*

eTable 4. Association of Modified Mediterranean Diet Score With Cognitive Performance at Visit 2

Cognitive Test	Mean scores (95% CI)			P value for trend	Mean differences, β (95% CI)	
	Low adherence group (n = 2020)	Moderate adherence group (n = 2741)	High adherence group (n = 1560)		Moderate vs. low adherence groups	High vs. low adherence groups
**Test Metrics**						
**B-SEVLT-Sum**						
Crude (n=6298)	22.15 (21.73-22.57)	22.88 (22.56-23.20)	23.55 (23.15-23.95)	<.001	0.72 (0.24-1.21)	1.40 (0.86-1.93)
Model 1[a] (n=6298)	21.83 (21.44-22.22)	22.77 (22.47-23.06)	23.44 (23.05-23.83)	<.001	0.93 (0.48-1.38)	1.61 (1.10-2.12)
Model 2[b] (n=6280)	21.71 (21.34-22.08)	22.62 (22.34-22.89)	23.52 (23.16-23.89)	<.001	0.90 (0.48-1.32)	1.81 (1.33-2.30)
Model 3[c] (n=5688)	22.64 (21.76-23.52)	23.20 (22.42-23.97)	23.83 (22.95-24.71)	<.001	0.55 (0.15-0.96)	1.20 (0.73-1.67)
**B--SEVLT-Recall**						
Crude (n=6286)	7.93 (7.71-8.15)	8.24 (8.08-8.40)	8.65 (8.42-8.87)	<.001	0.31 (0.05-0.58)	0.72 (0.42-1.02)
Model 1[a] (n=6286)	7.78 (7.58-7.98)	8.19 (8.04-8.35)	8.60 (8.37-8.83)	<.001	0.41 (0.17-0.65)	0.82 (0.53-1.10)
Model 2[b] (n=6268)	7.74 (7.54-7.93)	8.14 (7.99-8.29)	8.63 (8.41-8.86)	<.001	0.40 (0.17-0.63)	0.89 (0.61-1.17)
Model 3[c] (n=5679)	8.10 (7.69-8.50)	8.38 (7.95-8.82)	8.75 (8.28-9.21)	<.001	0.29 (0.06-0.51)	0.66 (0.36-0.95)
**Word fluency**						
Crude (n=6276)	18.21 (17.77-18.64)	18.07 (17.68-18.46)	18.44 (17.91-18.97)	.53	-0.14 (-0.70-0.42)	0.23 (-0.42-0.88)
Model 1[a] (n=6276)	18.16 (17.73-18.59)	18.09 (17.71-18.48)	18.49 (17.94-19.03)	.47	-0.06 (-0.60-0.48)	0.32 (-0.34-0.97)
Model 2[b] (n=6258)	18.00 (17.64-18.37)	17.90 (17.53-18.27)	18.68 (18.18-19.18)	.03	-0.11 (-0.61-0.39)	0.70 (0.10-1.29)
Model 3[c] (n=5669)	20.03 (18.80-21.27)	19.99 (18.80-21.17)	20.42 (19.08-21.76)	.31	-0.05 (-0.56-0.46)	0.49 (-0.13-1.11)
**DSST**						
Crude (n=6234)	32.97 (32.00-33.93)	31.97 (31.20-32.75)	32.27 (31.20-33.35)	.23	-0.99 (-2.14-0.15)	-0.69 (-2.00-0.61)
Model 1[a] (n=6234)	32.55 (31.71-33.38)	31.98 (31.23-32.72)	32.36 (31.34-33.38)	.52	-0.59 (-1.61-0.44)	-0.13 (-1.33-1.07)
Model 2[b] (n=6218)	32.12 (31.40-32.83)	31.44 (30.77-32.11)	32.66 (31.80-33.51)	.03	-0.69 (-1.59-0.20)	0.62 (-0.37-1.62)
Model 3[c] (n=5638)	38.06 (36.40-39.73)	37.98 (36.27-39.70)	38.52 (36.76-40.29)	.50	-0.15 (-0.97-0.67)	0.58 (-0.41-1.56)
**Z-scored Metrics**						
**B-SEVLT-Sum**						
Crude (n=6298)	-0.16 (-0.23-(-)0.09)	-0.03 (-0.09-0.02)	0.08 (0.01-0.15)	<.001	0.12 (0.04-0.21)	0.24 (0.15-0.33)

© 2022 Moustafa B et al. *JAMA Network Open.*

Model 1[a] (n=6298)	-0.21 (-0.28-(-)0.15)	-0.05 (-0.10-(-)0.002)	0.06 (-0.004-0.13)	<.001	0.16 (0.08-0.24)	0.28 (0.19-0.37)
Model 2[b] (n=6280)	-0.23 (-0.30-(-)0.17)	-0.08 (-0.13-(-)0.03)	0.08 (0.01-0.14)	<.001	0.15 (0.08-0.23)	0.31 (0.23-0.40)
Model 3[c] (n=5688)	-0.07 (-0.23-0.08)	0.02 (-0.11-0.15)	0.13 (-0.02-0.28)	<.001	0.10 (0.03-0.17)	0.22 (0.140.30)
**B--SEVLT-Recall**						
Crude (n=6286)	-0.16 (-0.24-(-)0.09)	-0.06 (-0.11-(-)0.01)	0.08 (0.00-0.15)	<.001	0.10 (0.02-0.19)	0.24 (0.14-0.34)
Model 1[a] (n=6286)	-0.21 (-0.28-(-)0.15)	-0.08 (-0.13-(-)0.02)	0.06 (-0.02-0.14)	<.001	0.14 (0.06-0.22)	0.27 (0.18-0.37)
Model 2[b] (n=6268)	-0.23 (-0.29-(-)0.16)	-0.09 (-0.14-(-)0.05)	0.07 (-0.004-0.15)	<.001	0.13 (0.06-0.21)	0.29 (0.20-0.39)
Model 3[c] (n=5679)	-0.11 (-0.24-0.03)	-0.01 (-0.16-0.13)	0.11 (-0.05-0.27)	<.001	0.09 (0.01-0.17)	0.22 (0.12-0.33)
**Word fluency**						
Crude (n=6276)	0.02 (-0.04-0.08)	-0.0004 (-0.05-0.05)	0.05 (-0.02-0.12)	.53	-0.02 (-0.10-0.06)	0.03 (-0.06-0.12)
Model 1[a] (n=6276)	0.01 (-0.05-0.07)	0.003 (-0.05-0.06)	0.06 (-0.02-0.13)	.47	-0.01 (-0.08-0.07)	0.04 (-0.05-0.13)
Model 2[b] (n=6258)	-0.01 (-0.06-0.04)	-0.02 (-0.07-0.03)	0.08 (0.01-0.15)	.03	-0.02 (-0.08-0.05)	0.09 (0.02-0.18)
Model 3[c] (n=5669)	0.27 (0.10-0.44)	0.26 (0.10-0.42)	0.32 (0.14-0.51)	.31	0.01 (-0.07-0.08)	0.08 (-0.01-0.17)
**DSST**						
Crude (n=6234)	.04 (-0.04-0.11)	-0.04 (-0.10-0.02)	-0.02 (-0.10-0.06)	.23	-0.08 (-0.16-0.01)	-0.05 (-0.15-0.05)
Model 1[a] (n=6234)	.004 (-0.06-0.07)	-0.04 (-0.10-0.02)	-0.01 (-0.09-0.07)	.52	-0.05 (-0.12-0.03)	-0.01 (-0.10-0.08)
Model 2[b] (n=6218)	-0.03 (-0.08-0.03)	-0.08 (-0.13-(-)0.03)	0.01 (-0.05-0.08)	.03	-0.05 (-0.12-0.02)	0.04 (-0.03-0.12)
Model 3[c] (n=5638)	0.42 (0.30-0.55)	0.42 (0.29-0.55)	0.46 (0.32-0.59)	.50	-0.01 (-0.07-0.06)	0.06 (-0.02-0.13)
**Global Cognition**						
Crude (n=6298)	-0.07 (-0.13-(-)0.01)	-0.04 (-0.08-0.01)	0.04 (-0.01-0.10)	.004	0.03 (-0.03-0.10)	0.12 (0.05-0.19)
Model 1[a] (n=62989)	-0.11 (-0.16-(-)0.05)	-0.04 (-0.08-(-)0.003)	0.04 (-0.02-0.10)	<.001	0.06 (0.002-0.12)	0.15 (0.08-0.22)
Model 2 [b] (n=6280)	-0.13 (-0.17-(-)0.08)	-0.07 (-0.11-(-)0.03)	0.06 (0.01-0.11)	<.001	0.06 (0.003-0.11)	0.18 (0.13-0.24)
Model 3 [c] (n=5688)	0.12 (0.01-0.24)	0.17 (0.06-0.27)	0.25 (0.13-0.37)	<.001	0.05 (-0.01-0.09)	0.14 (0.08-0.21)

Abbreviations: B-SEVLT, Brief -Spanish- English Verbal Learning Test; DSST, Digit Symbol Substitution Test.

[a]Adjusted by age and sex.

[b]Adjusted by age, sex, and educational level.

[c]Adjusted by age, sex, educational level, language preference, history of hypertension, history of stroke or transient ischemic attack, current or previous smoking status, health insurance, household income, US born, physical activity, kidney function, body mass index, and number of vascular risk factors.

© 2022 Moustafa B et al. *JAMA Network Open.*

eTable 5. Cognitive Performance Change Between Visits 1 and 2 by Modified Mediterranean Diet Adherence

Cognitive Test	Mean scores (95% CI)			P value for trend	Mean differences, β (95% CI)	
	Low adherence group (n = 2020)	Moderate adherence group (n = 2741)	High adherence group (n = 1560)		Moderate vs. low adherence groups	High vs. low adherence groups
**Test Metrics**						
**B-SEVLT-Sum**						
Crude (n=6266)	-.33 (-.64-(-).01)	.18 (-.08-.44)	.31 (-.02-.64)	.006	0.51 (0.15-0.87)	0.62 (0.19-1.06)
Model 1[a] (n=6266)	-.52 (-.83-(-).21)	.12 (-.13-.38)	.33 (.0001-.65)	<.001	0.64 (0.29-0.99)	0.83 (0.40-1.26)
Model 2[b] (n=6248)	-.59 (-.90-(-).28)	.06 (-.19-.31)	.44 (.11-.76)	<.001	0.65 (0.30-1.0)	1.00 (0.57-1.43)
Model 3[c] (n=5628)	-.18 (-1.06-.70)	.28 (-.50-1.07)	.52 (-.38-1.43)	.003	0.46 (0.12-0.80)	0.70 (0.27-1.11)
**B--SEVLT-Recall**						
Crude (n=6258)	-.18 (-.36-.01)	.04 (-.09-.18)	.13 (-.04-.31)	.051	0.22 (-0.01-0.45)	0.29 (0.04-0.54)
Model 1[a] (n=6258)	-.28 (-.45-(-).10)	.01 (-.12-.15)	.15 (-.03-.34)	.002	0.29 (0.08-0.50)	0.40 (0.15-0.65)
Model 2[b] (n=6240)	-.30 (-.48-(-).12)	-.01 (-.14-.12)	.20 (.01-.38)	<.001	0.29 (0.08-0.50)	0.46 (0.21-0.71)
Model 3[c] (n=5623)	-.31 (-.68-.05)	-.07 (-.47-.33)	.07 (-.35-.49)	.01	0.24 (0.03-0.45)	0.39 (0.13-0.65)
**Word fluency**						
Crude (n=6183)	-.16 (-.53-.22)	-.43 (-.68-(-).18)	-.74 (-1.08-(-).39)	.07	-0.27 (-0.72-0.19)	-0.58 (-1.08-(-)0.07)
Model 1[a] (n=6183)	-.16 (-.53-.21)	-.40 (-.65-(-).15)	-.67 (-1.02-(-).33)	.12	-0.24 (-0.69-0.21)	-0.51 (-1.01-(-)0.01)
Model 2[b] (n=6166)	-.21 (-.56-.13)	-.45 (-.71-(-).19)	-.55 (-.90(-)-.20)	.39	-0.24 (-0.68-0.19)	-0.28 (-0.78-0.22)
Model 3[c] (n=5565)	.56 (-.31-1.42)	.48 (-.33-1.30)	.29 (-.61-1.19)	.53	-0.07 (-0.51-0.38)	-0.25 (-0.77-0.27)
**DSST**						
Crude (n=6093)	-2.07 (-2.57-(-)1.57)	-2.22 (-2.62-(-)1.83)	-2.43 (-2.88-(-)1.98)	.54	-0.15 (-0.80-0.49)	-0.35 (-0.99-0.30)
Model 1[a] (n=6093)	-2.14 (-2.63-(-)1.65)	-2.24 (-2.62-(-)1.87)	-2.38 (-2.82-(-)1.93)	.77	-0.12 (-0.73-0.49)	-0.23 (-0.88-0.41)
Model 2[b] (n=6093)	-2.25 (-2.73-(-)1.78)	-2.42 (-2.80-(-)2.05)	-2.34 (-2.79-(-)1.88)	.85	-0.18 (-0.78-0.41)	-0.09 (-0.71-0.54)
Model 3[c] (n=5508)	-1.83 (-3.03-(-).64)	-1.95 (-3.15-(-).75)	-2.02 (-3.25-(-).80)	.84	-0.13 (-0.75-0.48)	-0.20 (-0.83-0.44)
**Z-scored Metrics**						
**B-SEVLT-Sum**						
Crude (n=6266)	-0.06 (-0.12-(-)0.01)	0.02 (-0.02-0.07)	0.05 (-0.01-0.10)	.006	0.09 (0.03-0.15)	0.11 (0.03-0.18)

© 2022 Moustafa B et al. *JAMA Network Open.*

Model 1[a] (n=6266)	-0.10 (-0.15-(-)0.04)	0.01 (-0.03-0.06)	0.05 (-0.01-0.10)	<.001	0.11 (0.05-0.17)	0.14 (0.07-0.22)
Model 2[b] (n=6248)	-0.11 (-0.16-(-)0.05)	0.0001 (-0.04-0.05)	0.07 (0.01-0.12)	<.001	0.11 (0.05-0.17)	0.17 (0.10-0.25)
Model 3[c] (n=5628)	-0.04 (-0.19-0.11)	0.04 (-0.09-0.18)	0.08 (-0.07-0.24)	.003	0.08 (0.02-0.14)	0.12 (0.05-0.19)
**B-SEVLT-Recall**						
Crude (n=6258)	-0.05 (-0.11-0.01)	0.02 (-0.03-0.07)	0.05 (-0.01-0.11)	.051	0.07 (-0.004-0.15)	0.10 (0.01-0.18)
Model 1[a] (n=6258)	-0.09 (-0.14-(-)0.03)	0.01 (-0.04-0.06)	0.06 (-0.01-0.12)	.002	0.10 (0.03-0.17)	0.13 (0.05-0.22)
Model 2[b] (n=6240)	-0.09 (-0.15-(-)0.04)	0.0001 (-0.04-0.05)	0.07 (0.01-0.13)	<.001	0.10 (0.03-0.17)	0.15 (0.07-0.24)
Model 3[c] (n=5623)	-0.10 (-0.22-0.02)	-0.02 (-0.15-0.11)	0.03 (-0.11-0.17)	.01	0.08 (0.01-0.15)	0.13 (0.04-0.22)
**Word fluency**						
Crude (n=6183)	0.05 (0.001-0.10)	0.01 (-0.03-0.04)	-0.03 (-0.08-0.01)	.07	-0.04 (-0.10-0.03)	-0.08 (-0.15-(-)0.01)
Model 1[a] (n=6183)	0.05 (0.0001-0.10)	0.01 (-0.02-0.05)	-0.02 (-0.07-0.02)	.12	-0.03 (-0.09-0.03)	-0.07 (-0.14-0.0001)
Model 2[b] (n=6166)	0.04 (-0.01-0.09)	0.01 (-0.03-0.04)	-0.01 (-0.06-0.04)	.39	-0.03 (-0.09-0.03)	-0.04 (-0.11-0.03)
Model 3[c] (n=5565)	0.14 (0.03-0.26)	0.13 (0.02-0.25)	0.11 (-0.02-0.23)	.53	-0.01 (-0.07-0.05)	-0.03 (-0.11-0.04)
**DSST**						
Crude (n=6093)	0.001 (-0.04-0.03)	-0.02 (-0.04-0.01)	-0.03 (-0.06-0.001)	.54	-0.01 (-0.06-0.04)	-0.03 (-0.08-0.02)
Model 1[a] (n=6093)	-0.01 (-0.05-0.03)	-0.02 (-0.04-0.01)	-0.03 (-0.06-0.01)	.77	-0.01 (-0.06-0.04)	-0.02 (-0.07-0.03)
Model 2[b] (n=6093)	-0.02 (-0.05-0.02)	-0.03 (-0.06-0.0001)	-0.02 (-0.06-0.01)	.85	-0.01 (-0.06-0.03)	-0.01 (-0.05-0.04)
Model 3[c] (n=5508)	0.01 (-0.08-0.10)	0.01 (-0.09-0.10)	0.0001 (-0.09-0.09)	.84	-0.01 (-0.06-0.04)	-0.02 (-0.06-0.03)
**Global Cognition**						
Crude (n=6285)	-0.01 (-0.04-0.03)	0.01 (-0.02-0.03)	-0.02 (-0.05-0.01)	.44	0.02 (-0.03-0.06)	-0.01 (-0.06-0.03)
Model 1[a] (n=6285)	-0.02 (-0.06-0.01)	0.0001 (-0.02-0.03)	-0.01 (-0.04-0.02)	.38	0.03 (-0.01-0.07)	0.01 (-0.03-0.06)
Model 2[b] (n=6267)	-0.03 (-0.06-0.0001)	0.0001 (-0.03-0.02)	0.01 (-0.02-0.04)	.17	0.03 (-0.01-0.07)	0.04 (-0.01-0.08)
Model 3[c] (n=5645)	0.01 (-0.08-0.10)	0.04 (-0.05-0.13)	0.04 (-0.06-0.14)	.25	0.03 (-0.01-0.07)	0.03 (-0.02-0.08)

Abbreviations: B-SEVLT, Brief -Spanish- English Verbal Learning Test; DSST, Digit Symbol Substitution Test.
[a]Adjusted by age and sex.
[b]Adjusted by age, sex, and educational level.
[c]Adjusted by age, sex, educational level, language preference, history of hypertension, history of stroke or transient ischemic attack, current or previous smoking status, health insurance, household income, US born, physical activity, kidney function, body mass index, and number of vascular risk factors.

© 2022 Moustafa B et al. *JAMA Network Open.*
